# Late-onset Bipolar I Disorder

**DOI:** 10.7759/cureus.3242

**Published:** 2018-08-31

**Authors:** Anthony Salem, Nikita Shah, Danielle Geraldi-Samara, Natarajan Elangovan, Michael Krzyzak

**Affiliations:** 1 Medicine, Staten Island University Hospital, Staten Island, USA; 2 Psychiatry, Staten Island University Hospital, Staten Island, USA; 3 Neurology, Staten Island University Hospital, Staten Island, USA

**Keywords:** bipolar mania, bipolar disorder, confusion

## Abstract

Bipolar I disorder is a chronic psychiatric illness in which patients alternate between symptoms of both mania and depression. Most cases are diagnosed when patients are 20 to 50 years old. We describe a patient presenting with symptoms of acute mania at an unusually late age for this condition. After a workup for an organic etiology, including imaging studies, this 71-year-old man was diagnosed with bipolar I disorder. Since diagnosis, his condition has been managed with risperidone.

## Introduction

Bipolar I disorder is a chronic psychiatric illness that causes patients to cycle through symptoms of mania to symptoms of depression and back again. Mania is defined as a distinct period of abnormally, persistently elevated, expansive, or irritable mood, and abnormally and persistently increased activity or energy, with symptoms lasting at least one week and present for most of the day, nearly every day (or any duration if hospitalization is necessary) [1]. Bipolar I disorder has a lifetime prevalence of 2.1% [2]. The National Alliance on Mental Illness reports that the average age-of-onset of bipolar disorder is about 25 years; however, it can occur earlier, presenting in childhood [3]. We present an unusual case of bipolar disorder emerging in a geriatric patient with no apparent organic causes.

## Case presentation

A 71-year-old Korean man presented from home with his family members for episodes of agitation, delusions, and confusion occurring intermittently over the past few weeks. The patient had no history of head injury, neck pain, recent stressors, travel or new medication. His past medical history was significant for diabetes, which was managed by lifestyle modifications. Family history was negative for any psychiatric history. At the time of the interview, the patient denied any headaches, chest pain, weight loss, abdominal pain, or dysuria. According to the patient's family, his behavior changed over the last three weeks and was marked with “making things up,” where he made bizarre, grandiose statements that he was a "billionaire" and a "lawyer,” among other inaccurate statements. The family also reported the patient underwent a personality change with increased irritability, aggressive outbursts towards neighbors and family (e.g., he punched a family member), and gathering/saving trash. Further, his family also reported episodes of forgetfulness along with his personality change.

**Figure 1:**
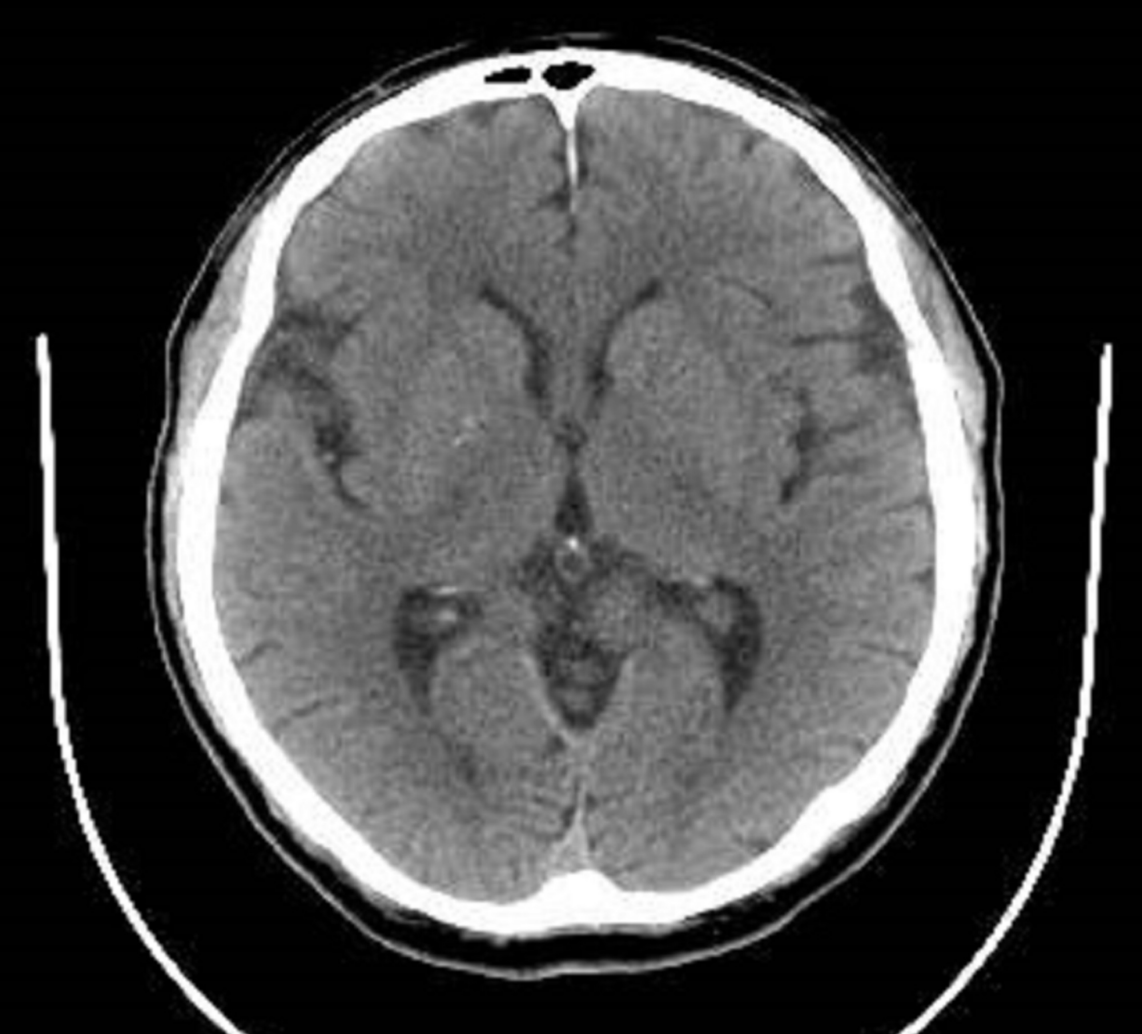
Magnetic resonance imaging of the head

**Figure 2:**
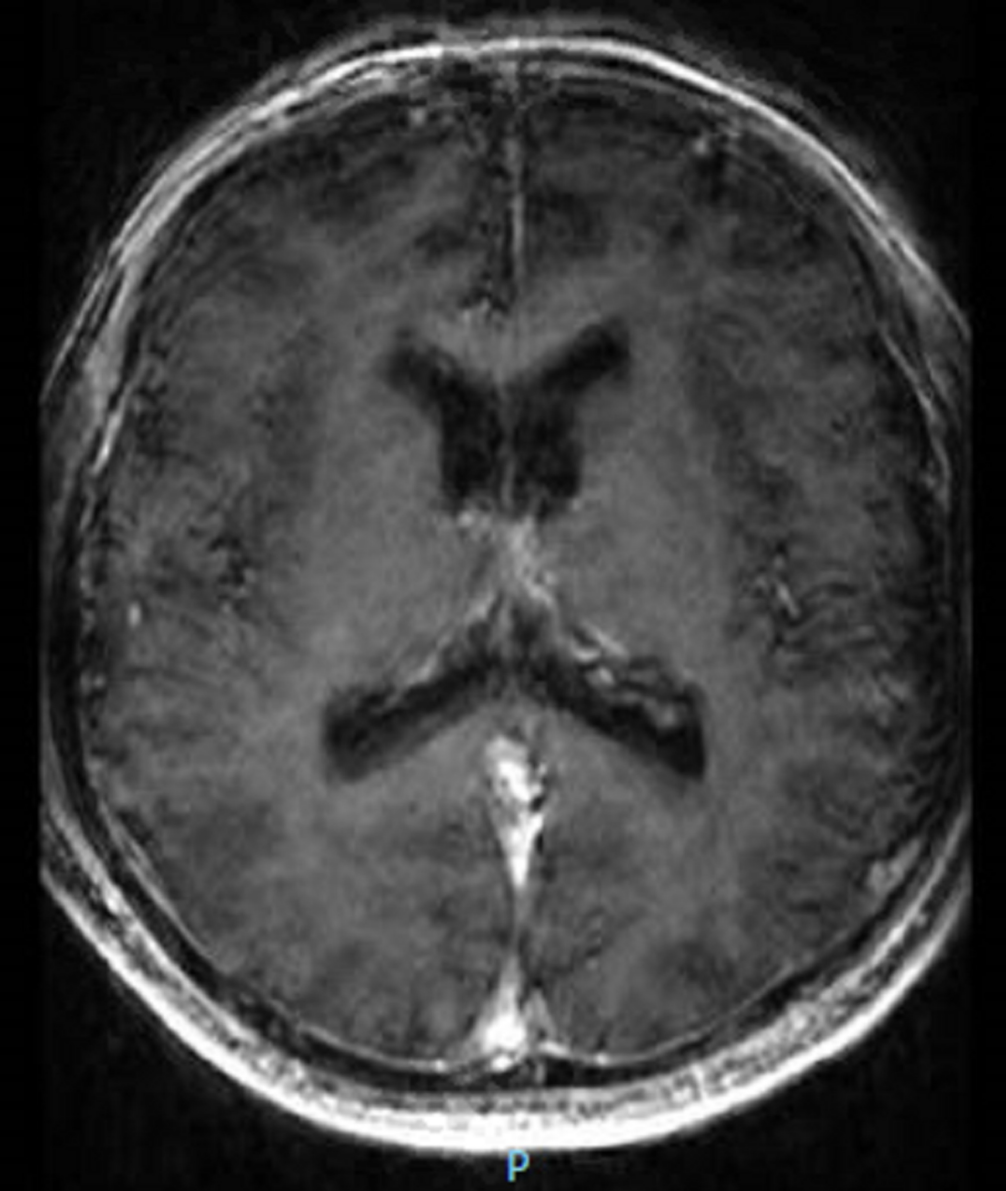
Computed tomography scan of the head

On initial examination, the patient had no insight; he stated that he had no psychiatric problems, that he was “richer than Bill Gates,” and that he could “buy a new house every month.” The patient also stated that his family did not understand him, and he would have to move to California where he has “many friends.” It is notable that, despite these claims, he oriented to time, place, and person during the interview. Upon initial assessment, the patient was admitted to the medical floor to rule out any underlying medical condition given his age at presentation as well as the sudden onset of symptoms. Clinical laboratory assessments included a drug screen, N-methyl-DA receptor antibodies, vitamin B12, folate, and syphilis screen along with thyroid studies, comprehensive metabolic panel, and complete blood count. The results of all laboratory assessments were either normal or within reference ranges. Radiologic studies performed included a computerized tomography (CT) scan and magnetic resonance imaging (MRI) of the patient’s head. The MRI showed a signal abnormality in the cerebral hemispheric white matter consistent with a chronic microvascular change (Figure 1). The CT scan showed no acute abnormalities (Figure 2). The psychiatry consultation-liaison followed the patient during medical admission. Although the presentation was consistent with bipolar disorder, the psychiatry consultation-liaison recommended a medical evaluation. Once the patient was medically cleared, the patient was started on aripiprazole and transferred to the inpatient psychiatric unit for further assessment and treatment.

Upon admission to the inpatient psychiatric unit, the patient appeared to respond well to aripiprazole with a reduction in symptoms. Therefore, his dose was increased. After the patient reported concerns of poor sleep, we started him on trazodone. However, within the first few days of his hospital course, the patient became increasingly delusional, grandiose, and threatening towards others. As his symptoms did not resolve over the next few days, the patient’s aripiprazole dose was reduced and then discontinued. The patient was then started on risperidone. As recommended by the neurology consultation, his care team performed a diagnostic lumbar puncture, which revealed no pathological findings. On risperidone, the patient’s mood continued to appear stable, his delusions lessened in intensity, and he functioned well on the unit. The patient was discharged home with follow-up instructions at an outpatient psychiatric care center.

## Discussion

Bipolar I disorder is a chronic illness characterized by cycles of depression and mania. A patient’s mood during manic episodes is reported as euphoric, excessively cheerful, high, or “feeling on top of the world" [1]. Late-onset bipolar disorder is classified according to two predisposing conditions. The first is late-life manic episodes in patients whose bipolar illness began during young adulthood, and the second is no prior manic episodes before late-life age but might have a history of depression. Most cases of bipolar I disorder are diagnosed in patients aged 20 to 50 years, and in 90% of the cases diagnosed, the patients are under the age of 50 [4]. Our case is unique given the late onset of symptoms, prompting a search for secondary reasons for his presentation. Despite a clean medical workup, our patient was diagnosed with bipolar I disorder.

## Conclusions

Clinicians should be aware of late-onset diseases that fall outside the common age range. Also, further investigation of this topic may provide evidence-based guidelines for treating late-onset bipolar disorder. Many patients in the geriatric population have various medical problems and medications and thus have increased risk factors that need to be carefully considered when choosing treatment options.
